# Subregional differences in the generation of fast network oscillations in the rat medial prefrontal cortex (mPFC) *in vitro*

**DOI:** 10.1113/JP270811

**Published:** 2015-06-03

**Authors:** Vasileios Glykos, Miles A Whittington, Fiona E N LeBeau

**Affiliations:** 1 Institute of Neuroscience, Newcastle University, Medical SchoolFramlington Place, Newcastle-upon-Tyne, NE2 4HH, UK; 2 York-Hull Medical School, F1- Department of Biology, York UniversityHeslington, YO10 5DD, UK

## Abstract

Fast network oscillations in the beta (20–30 Hz) and low gamma (30–80 Hz) range underlie higher cognitive functions associated with the medial prefrontal cortex (mPFC) including attention and working memory. Using a combination of kainate (KA, 200 nm) and the *cholinergic* agonist carbachol (Cb, 10 μm) fast network oscillations, in the beta frequency range, were evoked in the rat mPFC *in vitro*. Oscillations were elicited in the prelimbic (PrL), infralimbic (IL) and the dorsopeduncular (DP) cortex, with the largest oscillations observed in DP cortex. Oscillations in both the PrL and DP were dependent, with slightly different sensitivities, on γ-aminobutyric acid (GABA)_A_, α-amino-3-hydroxy-5-methyl-4-isoxazolepropionic acid (AMPA) and kainate receptors, but only oscillations in the DP were significantly reduced by *N*-methyl-d-aspartate (NMDA) receptor blockade. Intracellular recordings showed that 9/20 regular spiking (RS) cells in the PrL exhibited a notable cAMP-dependent hyperpolarisation activated current (*I*_h_) in contrast to 16/17 in the DP cortex. Extracellular single unit recordings showed that the majority of cells in the PrL, and DP regions had interspike firing frequencies (IFFs) at beta (20–30 Hz) frequencies and fired at the peak negativity of the field oscillation. Recordings in DP revealed presumed inhibitory postsynaptic potentials (IPSPs) that were larger in amplitude and more rhythmic than those in the PrL region. Our data suggest that each PFC subregion may be capable of generating distinct patterns of network activity with different cell types involved. Variation in the properties of oscillations evoked in the PrL and DP probably reflects the distinct functional roles of these different PFC regions.

**Key points**

- Fast network oscillations in the beta (20–30 Hz) frequency range can be evoked with combined activation of muscarinic and kainate receptors in different subregions of the medial prefrontal cortex (mPFC).
- Subregional differences were observed as the oscillations in the dorsal prelimbic cortex (PrL) were smaller in magnitude than those in the ventral dorsopeduncular (DP) region, and these differences persisted in trimmed slices containing only PrL and DP regions.
- Oscillations in both regions were dependent upon GABA_A_ and AMPA receptor activation but NMDA receptor blockade decreased oscillations only in the DP region.
- Subregional differences in neuronal properties of the presumed pyramidal cells were found between PrL and DP, with many more cells in DP firing rhythmically compared to the PrL region.
- Presumed inhibitory synaptic potentials (IPSPs) recorded from principal cells were more rhythmic and coherent, and significantly larger in amplitude, in the DP region; the data suggest that variation in the patterns of activity between subregions may reflect distinct functional roles.

## Introduction

In both humans and rodents the prefontal cortex (PFC) plays a key role in many higher executive functions including working memory, attention, decision-making, goal-directed behaviour and autonomic functions (Miller & Cohen 2001; Heidbreder & Groenewegen 2003; Seamans *et al*. 2008; Kesner & Churchwell 2011; Euston *et al*. 2012), while abnormalities in this region underlie many neuropsychiatric conditions such as drug addiction (Goldstein & Volkow 2011) and schizophrenia (Lewis *et al*. 2005, 2012; Uhlhaas *et al*. 2010, 2013). Network oscillations in the beta (20–30 Hz) and gamma (30–80 Hz) frequency are thought to be essential for many of the PFC functions (Benchenane *et al*. 2011; Roux *et al*. 2012). However, despite an important role for oscillations in the PFC we still know very little about the detailed cellular mechanisms that generate such activity in this region.

There is now considerable evidence to suggest that within the human, primate and rodent PFC different subregions mediate distinct cognitive functions (Ongur & Price 2000; Heidbreder & Groenewegen 2003; Seamans *et al*. 2008; Kesner & Churchwell 2011). In the rodent many studies now functionally distinguish the dorsal medial PFC (mPFC), which includes the anterior cingulate (ACC) and prelimbic (PrL) regions, and the ventral mPFC that includes the infralimbic (IL) and dorsopeduncular (DP) cortex. In particular, the dorsal regions of mPFC have been implicated in working memory and attentional tasks, while more ventral regions are linked to visceral and autonomic functions (Heidbreder & Groenewegen 2003). Underlying these functional differences are clear anatomical differences between the dorsal and ventral mPFC both in terms of efferent and afferent projections and cytoarchitecture (van Eden and Uylings 1985; Uylings *et al*. 2003; Heidbreder & Groenewegen 2003; Vertes 2004; Gabbott *et al*. 2005; Hoover & Vertes 2007; Akhter *et al*. 2014; Zinng *et al*. 2014).

We hypothesised that the different functional roles of the mPFC subregions could be subserved by differences in the network oscillations and/or neuronal properties in each region. A few studies have now demonstrated that fast network oscillations can be generated *in vitro* in the mPFC and these occur in the theta (8–15 Hz), beta (20–30 Hz) and slow gamma (30–80 Hz) frequency ranges (van Aerde *et al*. 2008, 2009; McNally *et al*. 2011, 2013; Steullet *et al*. 2014). However, these studies looked in separate regions and used different pharmacological methods to evoke activity, with either bath application of carbachol in PrL and IL of the rat (van Aerde *et al*. 2008, 2009) or pressure ejection of kainate in the PrL of mice (McNally *et al*. 2011), which limits our ability to compare activity directly across the functionally distinct regions of the mPFC.

The aim of this study was therefore to use a combination of carbachol and kainate (Cb–KA) to elicit stable persistent fast network oscillations in each subregion of the mPFC. In particular we focused on the dorsal mPFC (PrL) region and the previously uncharacterised ventral (DP) region. We found network oscillations were evoked in the beta (20–30 Hz) range that differed between dorsal and ventral mPFC with respect to oscillation magnitude, NMDA dependence, pyramidal cell properties and inhibitory inputs. These data support the idea that the cellular and network properties within mPFC subregions are capable of generating distinct patterns of oscillatory activity that could contribute to the increasingly delineated functional roles of the mPFC subregions described *in vivo*.

## Methods

### Animals and slice preparation

Male adult Lister Hooded rats were anaesthetised with inhaled isoflurane prior to intramuscular injection of ketamine (100 mg kg^−1^; Fort Dodge Animal Health Ltd, Southampton, UK) and xylazine (10 mg kg^−1^; Animalcare Ltd, York, UK). When all response to noxious stimuli, such as pedal withdrawal reflex, had terminated, the animals were intracardially perfused with ∼25 ml of modified artificial cerebrospinal fluid (ACSF) composed of (in mm): 252 sucrose, 3.0 KCl, 1.25 NaH_2_PO_4_, 24 NaHCO_3_, 2.0 MgSO_4_, 2.0 CaCl_2_ and 10 glucose. All procedures were in accordance with the UK Animals (Scientific Procedures) Act 1986 and the European Union Directive 2010/63/EU.

Following brain removal, 450 μm thick coronal PFC slices were cut using a Leica VT1000S vibratome. All slices were then transferred to either a holding chamber at room temperature, or a recording chamber where they were maintained at ∼29–31°C at the interface between normal ACSF (where sucrose was replaced with 126 mm NaCl and MgSO_4_ and CaCl_2_ were reduced to 1.2 and 1.76 mm, respectively) and humidified 95% O_2_ –5% CO_2_. In experiments where the anatomical separation between PrL and DP was required, a cut through the IL was performed in the recording chamber.

#### Drugs

Drugs used were as follows: Carbachol (10 μm), kainate (200 nm), (all from Sigma-Aldrich, Gillingham, Dorset, UK). Gabazine (1–10 μm), d-AP5 (100 μm), GYKI 52466 (50 μm) (all from Tocris, Bristol, UK).

### Experimental protocols

Oscillations were generated with the bath application of carbachol (Cb; 10 μm) and kainate (kainic acid, KA; 200 nm). Recording epochs of 60–120 s were taken at 5–15 min intervals and the power and frequency of the oscillations were extracted with power spectral analysis (see below). Stability of the oscillations was defined as being when the area power of three successive recordings at 10–15 min intervals deviated by < ±10%. At the end of the multi-electrode recordings, the array was inserted deeper into the slice for ∼15 min to mark the electrode position. The laminar and regional identification of the recording electrodes was confirmed with a Nissl staining procedure (see below).

### Electrophysiological recordings and data acquisition

#### Multi-electrode activity recordings

Local field potential and extracellular unit activity were recorded from multiple sites with a multi-electrode Utah array (Alpha Omega GmbH, Germany). The array was composed of 100 electrodes (10 rows × 10 columns) with inter-electrode distance of 400 μm and impedance ∼500 kΩ at 1 kHz. The array was inserted into the brain slice and remained in the same position throughout the course of the experiment. Analogue signals were recorded using the Cerebus-128 Front-End Amplifier (I2S Micro Implantable Systems, LLC, Salt Lake City, UT, USA). Signals were amplified, filtered (0.3 Hz–7.5 kHz) and digitized with a 16-bit resolution at a 30 kHz rate. Digital signals were transferred to the Cerebus-128 Neural Signal Processor. Further filtering separated the broadband digital signal into low (<250 Hz) and high (>250 Hz) frequency traces. The low frequency trace contained the local field potential signal that was down-sampled to a 2 kHz rate. The high frequency trace contained the fast spiking events.

On-line spike threshold detection was set manually. Once an extracellular spike exceeded the threshold, the spike waveforms (reconstructed by 48 samples) and the firing times (i.e. spike-timestamps) were extracted. Field potential signals, spike waveforms and timestamps recorded from every electrode, were transformed into a MATLAB (MathWorks Inc., Natick, MA, USA) compatible format and saved for further off-line analysis.

#### Single extracellular population field and intracellular recordings

Single extracellular field and intracellular recordings were made using a standard interface recording chamber. Extracellular recording electrodes were filled with normal ACSF (resistance 2–5 MΩ). Intracellular recording electrodes were made using 2 m potassium acetate-filled glass microelectrodes pulled to resistances of between 70 and 120 MΩ. Intracellular recordings from PFC neurons were performed in normal ACSF or in the presence of network oscillations. Only data recorded from cells with resting membrane potential of at least −50 mV and spikes exceeding +55 mV were used. IPSPs and excitatory postsynaptic potentials (EPSPs) were recorded at holding potentials of −30 mV and −70 mV, respectively.

Data were recorded with an Axoclamp-2B amplifier (Axon Instruments Inc., Union City, CA, USA). Extracellular data were filtered at 0.001–0.4 kHz and intracellular signals were low pass filtered at 2 kHz using Neurolog filters (Digitimer, Welwyn Garden City, Herts, UK). Mains noise was subtracted from the signal with a Humbug (Digitimer). Data were re-digitised at 10 kHz using an ITC-16 interface (Digitimer). Data were recorded using Axograph 4.6 software (Axon Instruments Inc.) and saved for further analysis.

### Data analysis

Unless otherwise stated, data analysis was performed with customised MATLAB software.

### Analysis of extracellular field and intracellular membrane potential recordings

#### Spectral analysis of extracellular field recordings

On-line power spectral analysis of population field activity was performed with Axograph (Axon Instruments). Off-line analysis was performed with customized MATLAB software. Accordingly, power spectral density (PSD) estimates were computed via the Welch's averaged modified periodogram method (0.25 Hz resolution). The area power of oscillations was calculated by integrating the area of the spectral power between 10 and 45 Hz. The predominant oscillation frequency was calculated from the frequency with the highest spectral power.

#### Rhythmicity extraction of extracellular field recordings

The rhythmicity index (RI) of network oscillations was determined from the normalized amplitude of the first side peak of the auto-correlations applied to the field trace.

#### Phase coherence analysis

Phase coherence analysis, initially introduced by Tass and colleagues (1998) was employed in the present study in order to assess (1) the dynamic interaction between two distant oscillating networks and (2) the contribution of excitatory or inhibitory synaptic input to the population field activity. The instantaneous phase was extracted from the extracellular field or intracellular membrane potential traces with the Hilbert transform. The mean phase difference and phase coherence were calculated with ‘CircStat’ a Matlab toolbox for circular statistics (Berens, 2009). Phase covariance was quantified with the log*Z* statistic, where *Z = R*^2^/*n* (*R =* resultant length, *n =* sample size). Histogram plots were also created to illustrate the phase covariance between the two oscillating systems. The probability mass function (pmf) in these plots was calculated by dividing the counts within each bin by the total number of counts.

### Detection and time extraction of spiking and synaptic events

The following paragraphs describe the techniques we applied to detect, isolate and extract the time of occurrence of individual spiking and synaptic events.

#### Spike detection and time extraction from intracellular recordings

Intracellular spikes were detected when positive membrane voltage deflections exceeded the mean baseline by more than 5 standard deviations. Visual inspection of data confirmed the spiking events. The exact time of the spiking events (spike-timestamps) was extracted and saved for further analysis.

#### Spike sorting analysis and time extraction of spiking events from extracellular multi-unit recordings

Extracellular single units were identified and isolated from multi-unit activity with a customized supervised *k*-means algorithm (i.e. point-to-cluster centroid Euclidean distance). Auto-correlograms were employed to confirm the isolation of single unit activity from multi-unit activity. Only units with a clean refractory period (i.e. no spiking events within <3 ms intervals) were included in the data analysis. The exact time of the spiking events (spike-timestamps) was extracted and saved for further analysis.

#### Detection and time extraction of IPSPs

IPSPs were recorded at ∼−30 mV in current-clamp conditions. The peaks and troughs within this periodic series of inhibitory synaptic events were extracted with the use of the first derivative of the voltage trace. The amplitude of the synaptic events was calculated by the voltage difference between the trough and the average amplitude of its preceding and succeeding peaks. Only voltage deflections larger than 1 mV were included in the data analysis. The time of occurrence of the maximal negative voltage deflection within the course of a single IPSP (IPSP-timestamp) was extracted and saved for further analysis.

### Data analysis of neuronal firing activity and synaptic input

The following paragraphs describe the analytical methods we applied to characterise the properties of neuronal firing activity and synaptic input from the spike- and IPSP-timestamps.

#### Average firing rate (AFR) of single cells

Neuronal firing activity was assessed with the average firing rate: 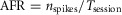, where *n*_spikes_ is the number of spikes the cell fired within the duration of the recording session, *T*_session_.

#### Neuronal rhythmicity index (NRI) analysis

The rhythmic properties of a single cell's firing activity (spike-timestamps) or synaptic input (IPSP-timestamps) were assessed with the same analytical tool, inspired by the spike ‘jittering’ method used in the identification of monosynaptic interactions by Fujisawa and colleagues (Fujisawa *et al*. 2008). First we produced 1000 surrogate data by jittering the original series of events by a uniform interval [−0.5, +0.5] s. Then, we produced the auto-correlograms of the original and the surrogate event series. All auto-correlograms were grouped into 3 ms bins within a range of [−2, 2] s. The envelopes of the positive-sided auto-correlograms were isolated and transformed into the frequency domain with the fast Fourier transform (FFT). The FFT amplitude values of the surrogate data were ranked in a descending order and the 99% confidence interval for each frequency point was produced (point-wise band). Then we calculated the point-wise ratio: 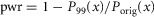, where *P*_99_ and *P*_orig_ are the FFT amplitudes of the 99% confidence interval and the original data, respectively, for every frequency point *x*. In cases where the ratio had negative values for all the frequency points, then the original series of events was considered non-rhythmic. Otherwise, the event series was considered rhythmic with a neuronal rhythmicity index (NRI) equal to the largest point-wise ratio. The prominent frequency of the periodic event series termed inter-spike firing frequency (IFF) was extracted from the frequency point with the highest ratio.

#### Neuronal phase coherence analysis

To assess the modulation of neuronal firing activity by the population field activity we extracted the instantaneous phase of the field trace coinciding with the spiking events. Phase extraction from the field trace was performed with the Hilbert transform. The mean firing phase, in addition to the phase modulation, were calculated with ‘CircStat’ in Matlab.

### Histology

Slices placed in buffered (4%) paraformaldehyde (PFA) solution for a varied period of 5 to 15 days. Slices were then removed from the PFA solution, mounted onto glass slides and dehydrated overnight at 4°C. Cortical tissue was Nissl stained with a Toluidine blue dye protocol. In brief the staining protocol included hydration by decreasing concentrations of ethanol (70%, 50%) for 1 min at each concentration, immersion in a Toluidine blue pH 0.5 dye solution for 7 min, dehydration by increasing concentrations of ethanol (50%, 70%, 90%, 100%) for 1 min and immersion in histoclear for 4 min. Coverslips were mounted on slides with histomount before microscope viewing. Images of the stained slices were taken from a microscope with magnification strength × 4 and × 10 and the laminar and regional position of electrodes was identified.

### Data grouping and statistical analysis

Data from the multi-electrode, single-electrode field or intracellular recordings were categorized into different groups with respect to the PFC region and layer. The normal distribution of data was assessed with the Kolmogorov–Smirnov normality test. When normality was accepted, data were described with mean and standard error of the mean (SEM) values. When normality failed, data were described with median and 25%–75% inter-quartile values (IQR).

To compare the differences between two unrelated groups of data, Student's unpaired *t*-test or the equivalent Mann–Whitney rank sum test were used for parametric or non-parametric data, respectively. To compare the differences between two related groups of data, Student's paired *t*-test or the equivalent Wilcoxon signed rank sum test were used for parametric or non-parametric data, respectively. To compare the differences between three or more unrelated groups of data, the one-way analysis of variance (ANOVA) or the equivalent Kruskal–Wallis one way analysis of variance on Ranks was used for parametric or non-parametric data, respectively. To compare the differences between three or more related groups of data, the one-way repeated measures (RM) ANOVA or the Friedman's RM ANOVA was used for parametric or non-parametric data, respectively. To measure the effect of the interaction of two independent variables on a dependent variable, the two-way ANOVA was used for parametric data. For phase values the circular equivalent Harrison–Kanji test was applied (Harrison & Kanji 1988). To measure the association strength and direction between two variables, the Pearson's product moment correlation was calculated.

To isolate the group or groups of data that differ significantly from the other, Tukey's multiple comparison test or Dunn's method was used. The difference between two groups of data was statistically significant when *P* < 0.05. Analysis of the circular statistics was performed with the ‘CircStat’ in a Matlab. The rest of the statistical analysis was made using SigmaStat (Systat Software Inc., San Jose, CA, USA).

## Results

### Subregional differences on network oscillations in mPFC

**Figure 1:**
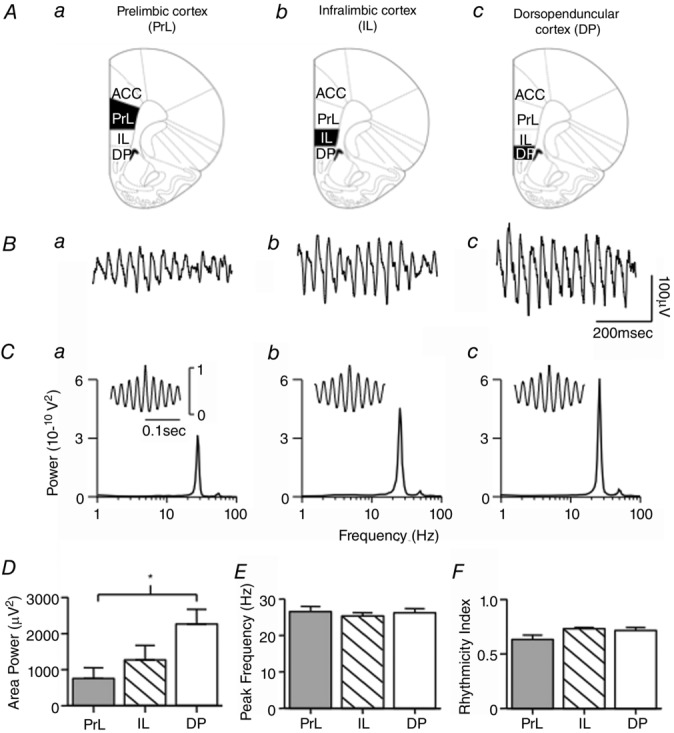
Comparison of fast network oscillations evoked in different subregions of the PFC *A*, schematic diagrams showing brain regions (black shading) used for prelimbic (PrL; *a*), infralimbic (IL; *b*) and dorsopeduncular (DP; *c*) cortex slices (interaural 11.70 mm). Adapted from Paxinos & Watson (2007). *B*, traces show 0.5 s epochs of extracellular field recordings showing fast network oscillations evoked by carbachol (10 μm) and kainate (200 nm) (Cb–KA) in the three subregions of mPFC. *C*, corresponding power spectra of 60 s of field recordings (insets show normalized autocorrelation of [−0.1–0.1] s). *D–F*, group data barplots of spectral and rhythmicity profiles of network oscillations in the mPFC. *D*, area power barplots show oscillations in PrL were significantly smaller than those in DP cortex (*P* < 0.05, one-way ANOVA, Tukey's test, *n* = 6). There was no significant difference in the frequency (*E*; *P* > 0.05, one-way ANOVA, *n* = 6) or the rhythmicity (*F*; *P* > 0.05, one-way ANOVA, *n* = 6) of the network oscillations between the three subregions.

In this study fast network oscillations were evoked in the mPFC (Fig.1) *in vitro* by bath application of a combination of 10 μm Cb and 200 nm KA (Cb–KA) following which oscillations increased in magnitude over the first 2–3 h of recordings as reported in hippocampus (Lu *et al*. 2012; Pietersen *et al*. 2014). Recordings were initially made in three different areas of the PFC, the prelimbic (PrL), infralimbic (IL) and dorsopeduncular (DP) regions. Activity was firstly recorded simultaneously across these three regions using a Utah multichannel array (Fig.1*A*) and the electrode locations (subregion and cortical layer) were confirmed by marking the electrode position and *post hoc* Nissl staining (see Methods).

Robust oscillations are not always recorded in every PFC slice; therefore, for the subsequent analysis the power of the oscillations was compared from slices in which stable oscillations were evident across cortical layers (III–VI) in all three regions (Fig.1*A–C*) of the PFC (PrL, IL and DP). Analysis of the control Cb–KA-evoked oscillations in layer V–VI revealed significant differences (Fig.1*D*), with the DP network producing the largest and the PrL network the smallest oscillations (PrL: 755 ± 299 μV^2^, IL: 1269 ± 401 μV^2^, DP: 2265 ± 407 μV^2^, *P* = 0.034, one-way ANOVA, Tukey's test, *n* = 6).

Fast network oscillations in all regions of the PFC were generally within the beta (20–30 Hz) range (Fig.1*E*) and there were no significant differences (PrL: 26.5 ± 1.4 Hz, IL: 25.3 ± 0.9 Hz, DP: 26.2 ± 1.2 Hz, *P* = 0.779, one-way ANOVA, *n* = 6) in frequency between any subregion. Autocorrelation analysis of the field traces (Fig.1*F*) showed that oscillations were highly rhythmic in all subregions (PrL: 0.63 ± 0.04, IL: 0.73 ± 0.01, D*P*: 0.72 ± 0.03, *P* = 0.066, one-way ANOVA, *n* = 6). As previously reported, beta frequency activity was also recorded in the ACC (Steullett *et al*. 2014) and motor cortex (Yamawaki *et al*. 2008) and in the tenia tecta (data not shown) but were not analysed further in this study.

**Figure 2:**
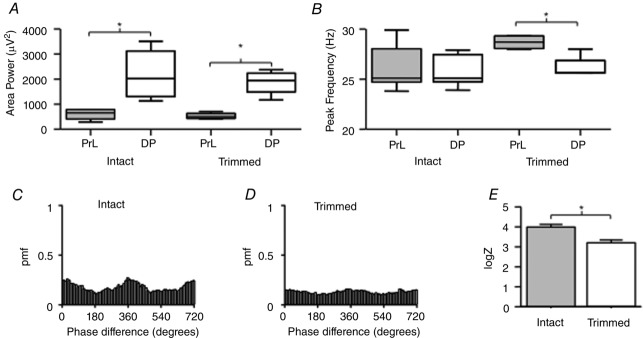
Subregional differences in network oscillations in trimmed PrL and DP slices Group data boxplots of the area power (*A*) and peak frequency (*B*) of network oscillations in the PrL and DP regions of intact and trimmed slices. The area power difference remained significant between PrL and DP regions after the two regions were anatomically separated with a cut across the IL (Intact: *P* < 0.05, Mann–Whitney rank sum test, *n*_PrL_ = 5, *n*_DP_ = 5, Trimmed: *P* < 0.05, Mann–Whitney rank sum test, *n*_PrL_ = 4, *n*_DP_ = 4). However, significant changes occurred in the peak frequency of network oscillations in the trimmed slices, with the PrL oscillating significantly faster than the DP (Intact: *P* > 0.05, Mann–Whitney rank sum test, *n*_PrL =_ 5, *n*_DP_ = 5, Trimmed: *P* < 0.05, Mann–Whitney rank sum test, *n*_PrL_ = 4, *n*_DP_ = 4). *C* and *D*, representative phase-coherence histograms (bin size: 10 deg) between layer V–VI oscillations recorded in the PrL and DP regions of intact (*C*) and trimmed (*D*) slices (2 cycles; 0–720 deg). *E*, group data barplot of the log*Z* values, showing that phase coherence between PrL and DP was significantly stronger in the intact slices (log*Z* values, intact *vs*. trimmed, *P* < 0.05, Student's unpaired *t* test, *n*_intact_ = 5, *n*_trimmed_ = 4) *Significant difference.

As one previous study has shown that oscillations in the PrL and IL can interact (van Aerde *et al*. 2008), we assessed oscillations in both PrL and DP in intact slices and in trimmed slices where the two regions of interest were anatomically separated with a lesion across the IL (Fig.2). Group data comparing dual field recordings from layer V–VI in intact slices from the PrL and DP demonstrated similar differences in area power between dorsal and ventral PFC as seen above with the Utah recordings (Fig.2*A*). Control area power in the PrL was smaller at 653 μV^2^ (IQR 406–782 μV^2^) *versus* 2019 μV^2^ (IQR 1300–3122 μV^2^) in the DP (*P* = 0.008, Mann–Whitney rank sum test, *n* = 5). In the trimmed slices this power difference remained as area power in the PrL was still smaller at 501 μV^2^ (IQR 433–627 μV^2^) *versus* 1946 μV^2^ (IQR 1480–2234 μV^2^) in the DP (*P* = 0.020, Mann–Whitney rank sum test, *n* = 4). Similarly as outlined above, there was no difference in the frequency of the oscillations between PrL and DP in the intact slices (Fig.2*B*), as in the DP slice the frequency was 25.1 Hz (IQR 24.7–27.4 Hz) compared to 25.1 Hz (IQR 24.7–28.0 Hz) in the PrL (*P* = 0.190, Mann–Whitney rank sum test, *n* = 5). In contrast, in the trimmed slices a frequency difference between PrL and DP emerged, with the frequency in the trimmed PrL slices significantly faster at 28.7 Hz (IQR 28.0–29.3 Hz) than the DP region at 25.6 Hz (IQR 25.6–26.8 Hz; PrL *P* = 0.029, Mann–Whitney rank sum test, *n* = 4). Finally, phase coherence analysis between parallel recordings in the deep layers of both regions revealed that network interaction was low in the intact slices and was significantly decreased further in trimmed slices (log*Z* values, intact: 3.99 ± 0.14, *n* = 5, trimmed: 3.22 ± 0.16, *n* = 4, *P* = 0.007, Student's unpaired *t* test, Fig.2*C–E*).

As the above data suggested a significant difference in the magnitude of the oscillations between the PrL and DP subregions of mPFC, we investigated network oscillations in these two regions in more detail particularly, as to our knowledge, oscillations in the DP region of the mPFC have not been previously reported.

### GABAergic and fast glutamatergic contributions to network oscillations in the PrL and DP regions

**Figure 3:**
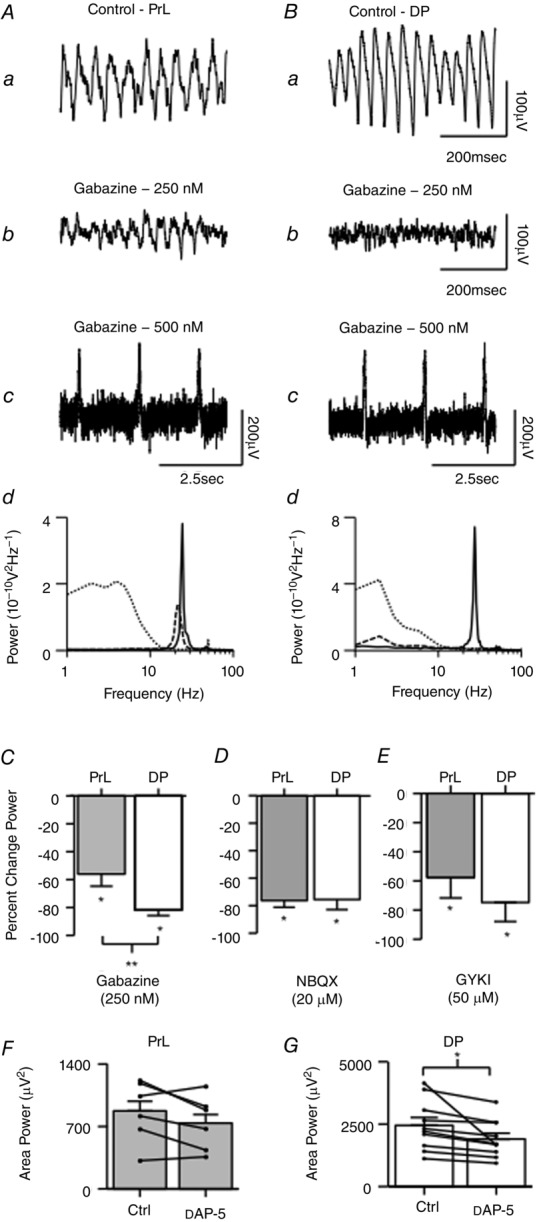
Role of GABA_A_, AMPA–kainate and NMDA receptor activation in the generation of fast network oscillations Traces show 0.5 s epochs of extracellular field recordings of oscillations in PrL (*A*) and DP (*B*) in control (*a*; Cb–KA), following bath application of the GABA_A_ receptor antagonist 250 nm gabazine (*b*), and following an increase to 500 nm gabazine (*c*). All oscillatory activity in both regions was abolished and slices showed burst discharges at a concentration of 500 nm gabazine. (*Ad* and *Bd, c*orresponding power spectra of 60 s recordings in control (continuous line) 250 nm gabazine (dashed line) and 500 nm gabazine (dotted line). *C*, histogram plot of percentage change in area power shows a significant reduction in the power of the oscillations in PrL and DP at 250 nm gabazine. The reduction was significantly greater in DP compared to PrL (*P* < 0.05, Student's unpaired *t* test, *n*_PrL_ = 9, *n*_DP_ = 9). *D*, histogram plot shows percentage reduction in the power of the oscillations in PrL and DP was similar in both regions with NBQX (10 μm). *E*, histogram plot of percentage change in area power shows a significant reduction in the power of the oscillations in both the PrL and DP regions with 10 μm GYKI. *F* and *G*, histogram plots show area power values during control and d-AP5 conditions in PrL and DP. A significant reduction was observed only in the DP region (*P* < 0.05, Student's paired *t* test, *n*_DP_ = 10). *Significant difference of area power values between control and drug conditions within each region. **Significant difference in the area power change between PrL and DP.

GABA_A_ receptor activation has been shown to be critical for the generation of network oscillations in the PrL, evoked either by Cb (van Aerde *et al*. 2008), or pressure ejection of KA (McNally *et al*. 2011). After obtaining a stable Cb–KA-evoked oscillation, bath application of the GABA_A_ receptor antagonist gabazine (250 nm) also reduced oscillatory activity in the PrL and DP regions in this study (Fig.3*A–C*). Control oscillation area power in the PrL was significantly reduced by 56 ± 9% (*P* = 0.001, Student's paired *t* test, *n* = 9). Oscillations in the DP were reduced by 82 ± 5% (*P* = 0.008, Student's paired *t* test, *n* = 9). Increasing the concentration of gabazine to 500 nm further reduced the oscillations in the PrL by 73 ± 7% (*P* < 0.001, Student's paired *t* test, *n* = 9) and in the DP by 91 ± 2% *P* = 0.007, Student's paired *t* test, *n* = 9). The difference in sensitivity to gabazine was significant between PrL and DP in both low (*P* = 0.032, Student's unpaired *t* test, *n* = 9) and high (*P* = 0.031, Student's unpaired *t* test, *n* = 9) gabazine concentrations. In 3/9 slices spontaneous epileptiform burst discharges were evident following 500 nm of gabazine application in both regions, occurring with a rate of 0.7 Hz (IQR 0.6–0.7 Hz) in the PrL (*n* = 3) and 0.6 Hz (IQR 0.5–0.6 Hz) in the DP (*n* = 3).

The contribution of fast phasic glutamatergic neurotransmission to network oscillations depends upon the cortical region of interest (Roopun *et al*. 2006; Yamawaki *et al*. 2008). Oscillations in motor cortex evoked by combined Cb–KA were not blocked by the AMPA receptor antagonist SYM 2206 (Yamawaki *et al*. 2008). We therefore investigated the role of fast glutamatergic neurotransmission firstly by bath applying the AMPA–kainate receptor antagonist NBQX (20 μm), which reduced all oscillatory activity (Fig.3*D*) by 76 ± 5.1% (*P* = 0.005, Student's paired *t* test, *n* = 7) in the PrL and by 75.3 ± 7.3% (*P* < 0.001, Student's paired *t* test, *n* = 8) in the DP (Fig.3*D*). Oscillations were reduced in area power in the PrL and DP regions by the specific AMPA receptor antagonist GYKI 52466 (50 μm; Fig.3*E*). The area power of the activity was reduced by 57.6 ± 9.9% (*P* = 0.005, Student's paired *t* test, *n* = 8) in the PrL and by 74.8 ± 8.3% and in the DP (*P* < 0.001, Student's paired *t* test, *n* = 10). The sensitivity to GYKI 52466 was not significantly different between the PrL and DP regions (*P* = 0.198, Student's unpaired *t* test).

The contribution of NMDA receptors to the generation of network oscillations also depends upon both the region assessed, and the method used to evoke oscillations, with increases, decreases or no change being reported following application of NMDA receptor antagonists (Fisahn *et al*. 1998; Cunningham *et al*. 2004; McNally *et al*. 2011). We therefore compared the effects of NMDA receptor blockade with d-AP5 (100 μm) on oscillations in PrL and DP (Fig.3*F* and *G*). In the PrL, bath application of d-AP5 had mixed effects on area power (Fig.3*F*). Overall a reduction of 12.9 ± 8.3% in the control oscillation area power was observed but this was not significantly different from control (*P* = 0.117; Student's paired *t* test, *n* = 6). The variability of the effect of d-AP5 in the PrL region was not correlated with the power of the initial control oscillations (*r* = −0.4, *P* = 0.437, Pearson's product moment correlation, *n* = 6). In contrast, in the DP region NMDA blockade caused a small but consistent reduction of 22 ± 4.9% in the power of the oscillations (Fig.3*G*) that was statistically significant (*P* = 0.034, Student's paired *t* test, *n* = 10).

There was no significant change in the frequency of the fast network oscillations in the presence of d-AP5 in either the PrL (control: 24.0 ± 1.0 Hz, d-AP5: 23.4 ± 0.7 Hz, *P* = 0.188, Student's paired *t* test, *n* = 6) or the DP region (control: 24.3 ± 0.7 Hz, d-AP5: 23.6 ± 0.6 Hz, *P* = 0.088, Student's paired *t* test, *n* = 10).

These data demonstrate that fast network oscillations in PrL and DP regions were similar in that both required activation of AMPA receptors. However, gabazine and d-AP5 both had a stronger effect in DP, suggesting a greater role of GABA_A_ inhibition and NMDA receptors in the generation of network activity in this region.

### Different neuronal subtypes in the PrL and DP regions

**Figure 4:**
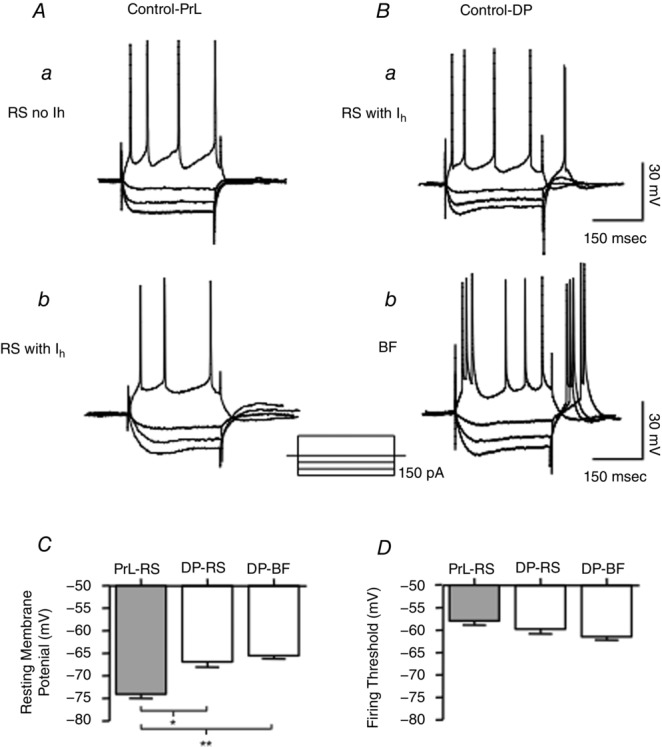
Intrinsic cell properties in the absence of network oscillations Membrane response of presumed pyramidal cells in PrL (*A*) and DP (*B*) regions to hyperpolarizing and depolarizing current pulses (300 ms, 50 pA current steps) in normal ACSF (no Cb–KA-evoked oscillations) of an RS cell without *I*_h_ (*Aa*) and an RS cell with *I*_h_ (*Ab*) in the PrL. *B*, membrane responses of an RS cell with *I*_h_ (*Ba*) and a BF cell in the DP under the same recording protocol as in *A* (*Bb*). *C*, barplots showing the significant difference in the resting membrane potential (*P* < 0.001, one-way ANOVA, Tukey's test, PrL: *n*_RS_ = 20, DP: *n*_RS_ = 9, *n*_BF_ = 8). *D*, barplots illustrating the mean firing threshold values (*P* > 0.05, one-way ANOVA, PrL: *n*_RS_ = 20, DP: *n*_RS_ = 9, *n*_BF_ = 8) for RS and BF cells in the mPFC.

Intracellular recordings from deep layer V–VI cells in the PrL and DP were first recorded in normal ACSF in the absence of Cb–KA (no-oscillation condition). Nearly all presumed pyramidal cells in PrL were regular spiking (RS) cells (20/21) with only one cell classified as having burst firing (BF) properties (Fig.4). As previously reported (Dembrow *et al*. 2010; Gee *et al*. 2012), we found that RS cells could be divided into two broad classes depending on whether a hyperpolarisation-activated cyclic nucleotide-gated cation (h) current (*I*_h_) was evident. Overall *I*_h_ was detected in 9/20 (45%) of RS cells in the PrL region (Fig.4*A*). In the DP region (Fig.4*B*) 9/17 (53%) cells were also classified as RS, 8 of which had an *I*_h_. DP also contained a large number of burst firing (BF) cells (8/17, 43%) all expressing an *I*_h_. Overall cells in the DP region had more depolarised resting membrane potentials (r.m.p.) and lower firing thresholds than cells in the PrL region (Fig.4*C* and *D*; Table 1), although the latter was not statistically significant.

**Table 1:** Intrinsic membrane properties of cells in the PrL and DP regions

Region	Cell	r.m.p. (mV)	Vthr (mV)
PrL	RS (*n* = 20)	−74.0 ± 1.0*,**	−58.0 ± 0.8
	BF (*n* = 1)	−70.0	−57.0
DP	RS (*n* = 9)	−67.0 ± 1.8*	−59.8 ± 1.6
	BF (*n* = 8)	−65.5 ± 1.2**	−61.5 ± 1.2

The table shows the mean ± SEM values of the resting membrane and firing threshold potentials of RS and BF cells in the PrL and DP. *,**Statistically significant difference between the r.m.p values of RS cells in the PrL and RS and BF cells in the DP (*P* < 0.001, one-way ANOVA, Tukey's test). DP cells tended to fire spikes at lower threshold. However, the difference in the mean firing threshold values of RS cells in the PrL and RS and BF cells in the DP was non-significant (*P* = 0.105, one-way ANOVA).

### Firing properties during fast network oscillations in PrL *versus* DP regions

To analyse the firing properties of cells in deep layers V–VI in the PrL and DP during Cb–KA-evoked network oscillations, we grouped data from both intracellular (*n*_PrL =_ 9, *n*_DP_ = 16) recordings with spike-sorted (see Methods) extracellular single unit (*n*_PrL =_ 42, *n*_DP_ = 29) recordings (*n*_total =_ 96 cells). It is important to note that in oscillating conditions the different intrinsic properties of cells become obscured and it is not possible to distinguish BF from RS cells so all cells were grouped together (Degenetais *et al*. 2002). When grouped together the majority of cells (93/96) fired spikes with a slow average firing rate < 10 Hz (median 3.8 Hz, IQR 2.7–5.8 Hz). Three cells had a fast average firing rate > 10 Hz (median 17.7 Hz, IQR 17.4–18.6 Hz). The slow firing cells probably correspond to putative pyramidal cells, whereas the fast firing cells may correspond to fast spiking interneurons. However, due to the small sample size, the fast spiking cells were excluded from the rest of the analysis. There was no significant difference in average firing rate for cells in PrL *versus* DP (PrL: median 4.3 Hz, IQR 2.6–5.8 Hz, *n* = 50, DP: median 3.7 Hz, IQR 2.7–5.8 Hz, *n* = 43, *P* = 0.997, Mann–Whitney rank sum test).

**Figure 5:**
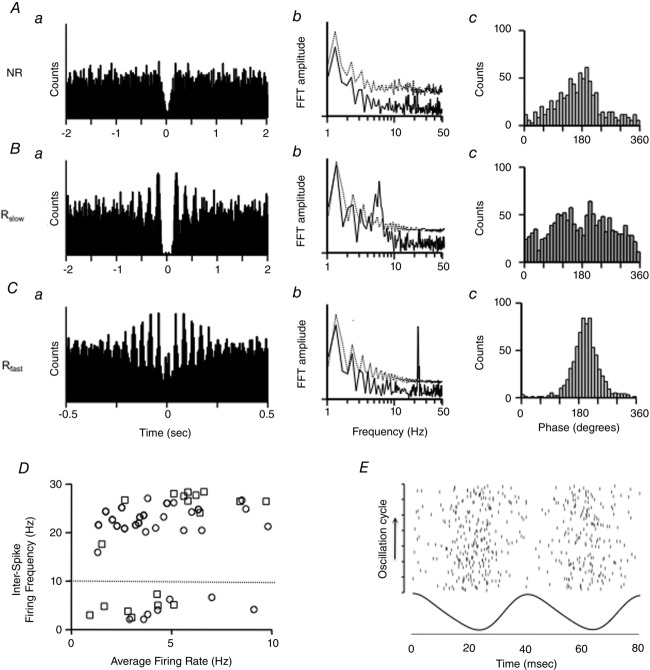
Rhythmic firing activity of single cells in the presence of Cb–KA-evoked network oscillations Examples of non-rhythmic NR (*A*), rhythmic and slow IFF (*B*; R_slow_) and rhythmic and fast IFF (*C*; R_fast_) cells. Corresponding autocorrelogram bin histograms (bin size: 3 ms) produced from the spike trains of individual cells are shown in *Aa–Ca*. *Ab–Cb*, semilogarithmic FFT amplitude plots were calculated from the [0–2] s range of the autocorrelogram envelope of the original and the 1000 jittered surrogates. Rhythmicity was extracted when the original FFT amplitude values (continuous line) exceeded in at least one frequency point the 99% of the ranked surrogates (dotted line). *Ac–Cc*, phase-coherence bin histograms (bin size: 10 deg) produced from the same spike trains as in *a* and *b*, showing the firing preference of the representative cells to a certain phase within the field cycle. *D*, scatterplot of the average firing rate against the inter-spike frequency of the rhythmic cells (PrL and DP units grouped together, *n* = 51), shows a clear separation between the R_slow_ and R_fast_ cells (below and above the dotted line, respectively). Squares represent PrL cells and circles represent DP cells. *E*, raster plot of the firing activity of the R_fast_ unit shown above in *C* during network oscillations, triggered by every second positive peak of the field trace. Continuous line is produced by averaging successive pairs of cycles from the field trace. The firing activity of the unit is preferably clustered around the peak negativity of the field trace.

We subsequently assessed the rhythmicity of the firing activity for every unit individually (see Methods) in the PrL and DP regions. Units were classified as rhythmic and non-rhythmic (NR; Fig.5) based on their neuronal rhythmicity index (NRI). Within the group of cells with rhythmic firing activity two sub-groups with different inter-spike firing frequencies (IFF) were observed. The first group of cells had slow (Fig.5*B*) IFF values < 10 Hz (R_slow_: median IFF 4.6 Hz, IQR 3.5–5.6 Hz, *n* = 14), whereas the second group had fast IFF values > 10 Hz (Fig.5*C*) within the beta frequency (20–30 Hz) band (R_fast_: median IFF Hz, 24.6 IQR 21.8–26.9 Hz, *n* = 37). Overall the majority of cells in the DP region (56%) were classified as R_fast_ cells (24/43), with 16% (7/43) classed as R_slow_ and 28% NR (12/43). In contrast in the PrL region the majority of cells (60%) were NR (30/50) with only 26% (13/50) classed as R_fast_ and 14% (7/50) classed as R_slow_ cells. The difference in the IFF values between PrL and DP cells within the R_slow_ group of cells was not statistically significant (PrL: median 5.3 Hz, IQR 3.7–5.6 Hz, *n* = 7, DP: median 4.5 Hz, IQR 2.9–6.2 Hz, *n* = 7, *P* = 0.620, Mann–Whitney rank sum test). However, within the R_fast_ group PrL cells fired with a slightly faster IFF (PrL: median 27.0 Hz, IQR 24.6–28.3 Hz, *n* = 13, DP: median 23.3 Hz, IQR 21.5–25.3 Hz, *n* = 24, *P* = 0.008, Mann–Whitney rank sum test). The firing pattern for one example R_fast_ unit (shown in Fig.5*C*) shows firing occurred on the peak negativity of the corresponding field oscillation (Fig.5*E*).

**Figure 6:**
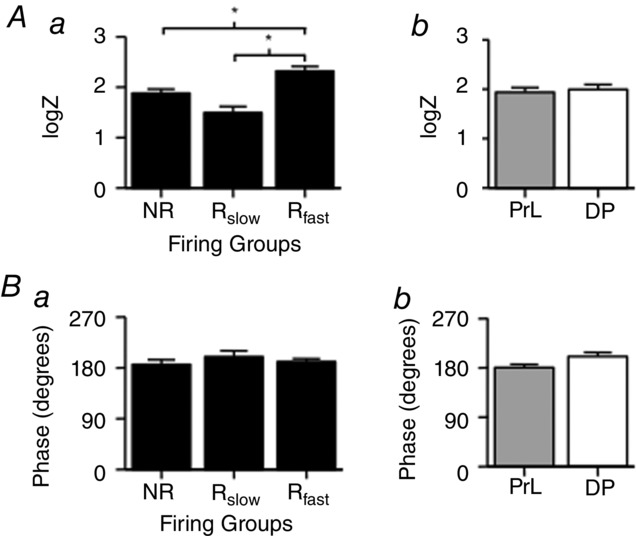
Phase coherence and phase preference of mPFC cell firing during Cb–KA-evoked network oscillations *A*, barplots of the phase-coherence log*Z* values grouped with respect to the rhythmicity profile cells exhibited (*a*; NR, R_slow_ or R_fast_) or the region they were recorded from (*b*; PrL or DP). Two-way ANOVA revealed a significant difference between cells with different rhythmicity profiles (*F*(2,88) = 9.550, *P* < 0.001, two-way ANOVA, Tukey's test, *n*_NR_ = 42, *n*_slow_ = 14, *n*_fast_ = 37). The difference between PrL and DP cells was not significant (*F*(1.88) = 0.03, *P* > 0.05, two-way ANOVA, *n*_PrL_ = 50, *n*_DP_ = 43). *B*, the same analysis as in *A* was performed on the phase values. Barplots show that mPFC cells with different rhythmicity profiles (*a*; *P* = 0.727, Harrison–Kanji test, *n*_NR_ = 42, *n*_slow_ = 14, *n*_fast_ = 37) or recorded from different regions (*b*; *P* = 0.281, Harrison–Kanji test, *n*_PrL_ = 50, *n*_DP_ = 43) fired spikes around the trough or the rising phase of the field cycle.

The engagement of single cells in the network oscillatory activity was assessed with the coherence value (log*Z*) of neuronal firing activity to the phase of the concurrently recorded field oscillation (Fig.6). We first assessed whether there was any difference in log*Z* values between NR, R_slow_ or R_fast_ units (Fig.6*A***)**. Two-way ANOVA revealed that the log*Z* value of R_fast_ cells was significantly higher compared to R_slow_ and NR cells (NR: 1.9 ± 0.1, *n* = 42, R_slow_: 1.5 ± 0.2, *n* = 14, R_fast_: 2.3 ± 0.1, *n* = 37, *P* < 0.001, Tukey's test, Fig.6*Aa*), but there was no difference between PrL and DP cells (PrL: 1.9 ± 0.1, *n* = 50, DP: 1.9 ± 0.1, *n* = 43, *P* = 0.873; Fig.6*Ab*), nor was there an interaction between the different regions and rhythmicity profiles *P* = 0.527).

All cells fired spikes at the peak negativity or rising phase of the periodic field cycle. The Harrison–Kanji circular analogue to the two-way ANOVA did not reveal any significant difference in the phase value of cells belonging to NR, R_slow_ and R_fast_ groups (NR: 194 ± 8, *n* = 42, R_slow_: 199 ± 13, *n* = 14, R_fast_: 188 ± 8, *n* = 37, *P* = 0.727, Fig.6*Ba*), or between cells in the PrL and DP regions (PrL: 187 ± 8, *n* = 50, DP: 201 ± 8, *n* = 43, *P* = 0.281, Fig.6*Bb*) and there was no interaction between the two main factors (*P* = 0.522).

### Different presumed IPSP properties between PrL and DP regions

**Figure 7:**
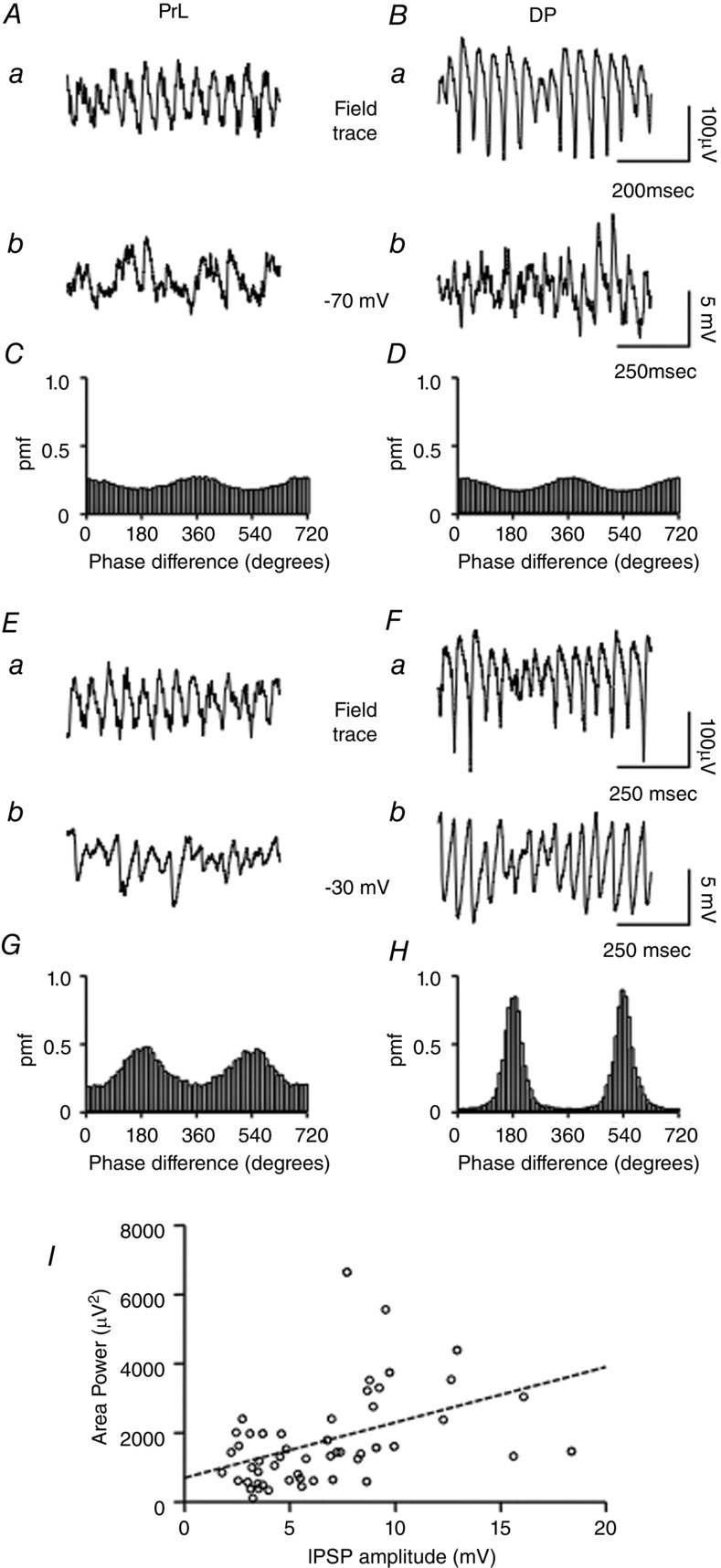
Regional differences of synaptic potentials in the mPFC Examples of extracellular field and synaptic activity recorded from presumed RS cells in PrL (*A*) and DP (*B*) during Cb–KA-evoked oscillations. *Aa* and *Ba*, 1 s example trace of extracellular field recording. *Ab* and *Bb*, 1 s epochs of EPSPs recorded at a membrane potential of −70 mV. Phase coherence histograms (bin size: 10 deg) between field and EPSPs in PrL (*C*) and DP (*D*) show low coherence. Different examples of extracellular field and synaptic activity recorded from presumed RS cells in (*E*) PrL and (*F*) DP during Cb–KA-evoked oscillations. *Ea* and *Fa*, 1 s example trace of extracellular field recording. *Eb* and *Fb*, 1 s epochs of IPSPs recorded at a membrane potential of −30 mV. Phase coherence histograms (bin size: 10 deg) between field and IPSPs in PrL (*G*) and DP (*H*) show high coherence. *I*, scatterplot showing a significant relationship between the IPSP amplitude of all mPFC cells (PrL and DP, *n* = 52) and the area power of the concurrently recorded population field activity; Pearson's product moment correlation (*r* = 0.45, *P* < 0.001). Dashed line represents linear regression.

In the hippocampus it has been suggested that the largest contribution to the extracellular field gamma frequency oscillation comes from synaptic inhibitory events (Oren *et al*. 2010). The larger area power field oscillations seen in the DP region in this study may, therefore, reflect differences in the inhibitory synaptic events occurring during the Cb–KA-evoked network oscillations in the PrL and DP regions. When recording in cells held at −70 mV, the postsynaptic potential (PSP) will consist largely of EPSPs. When recording at −30 mV, the PSP will be a mixed EPSP–IPSP, although the predominant component is most likely the IPSP, and we will refer to these PSPs recorded at −30 mV as ‘presumed IPSPs’ or IPSPs. PSPs were recorded from layer V–VI presumed pyramidal cells, held at −30 mV and −70 mV, respectively, once stable Cb–KA oscillations had been obtained (Fig.7). EPSPs were irregular and showed no coherence with the field oscillations (Fig.7*A–D*). In contrast rhythmic presumed IPSPs were recorded in both regions at −30 mV during oscillations (Fig.7*E–H*) and the phase difference histograms showed the IPSPs were in anti-phase to the field oscillations (∼180 deg). In addition, when IPSP amplitudes from both PrL and DP were grouped together there was a significant correlation with the power of the concurrently recorded field power (Fig.7*I*), suggesting that the IPSPs make the largest contribution to the field oscillation (Pearson product moment correlation *r* = 0.45, *P* < 0.001).

**Figure 8:**
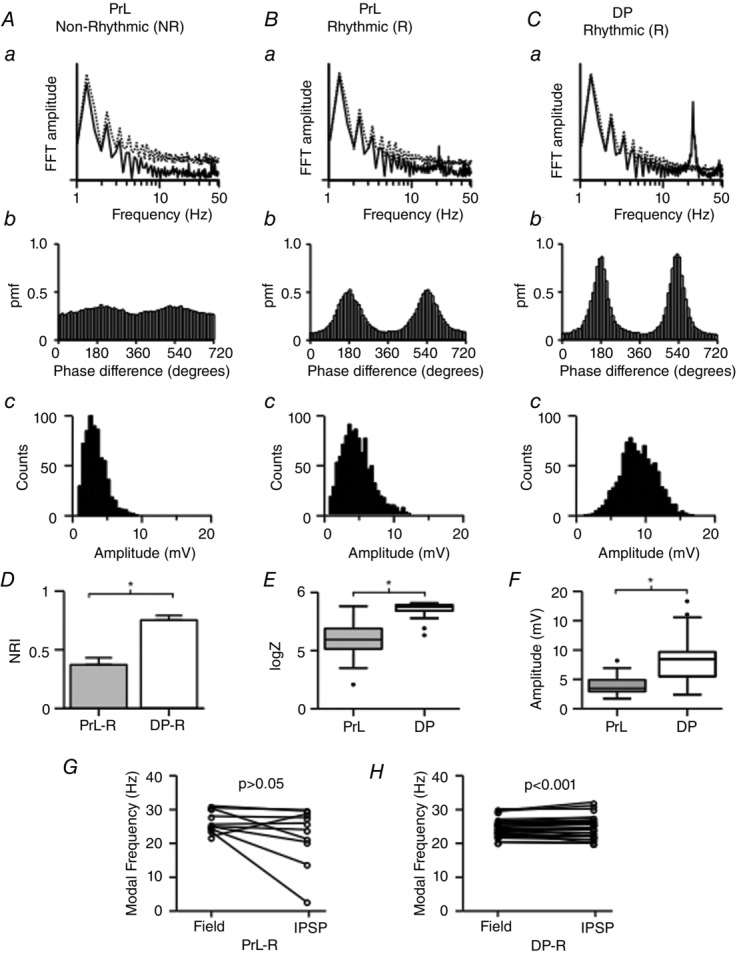
Distinct regional differences in IPSP properties Two different patterns of inhibitory synaptic activity were recorded from the mPFC cells. *A*, in 10/22 PrL cells, IPSPs exhibited non-rhythmic properties (PrL-Non Rhythmic; PrL-NR). *B*, 12/22 PrL cells received rhythmic inhibitory synaptic input (PrL-Rhythmic; PrL-R). *C*, in contrast, in the DP region all cells (30/30) received rhythmic IPSPs (DP-Rhythmic; DP-R). *Aa–Ca*, semilogarithmic FFT amplitude plots of the original trace (continuous traces) and the 99% confidence interval of the 1000 jittered surrogates (dotted traces). *Ab–Cb*, phase-coherence histograms (bin size: 10 deg) produced from IPSP traces and concurrently recorded population field traces. *Ac–Cc*, representative bin histograms of the amplitude of IPSP events (bin size: 0.5 mV) extracted from 60 s traces. *D*, barplots of the NRI values for all cells in (PrL *n* = 22, DP *n* = 30, *P* < 0.001, Student's unpaired *t* test). *E*, phase-coherence log*Z* boxplots showing that the phase coherence of the DP IPSPs was significantly stronger to the population field trace compared to the PrL IPSPs (*P* < 0.001, one-way ANOVA, Tukey's test, PrL *n* = 22, DP *n* = 30). *F*, boxplots showing the significant difference between the IPSP amplitude of DP and PrL IPSPs (*P* < 0.001, one-way ANOVA, Tukey's test, PrL *n* = 22, DP *n* = 30). The modal frequencies of the field and the IPSP traces are plotted together for the PrL-R (*G*) and the DP-R (*H*) groups. The relationship between the field and IPSP frequencies was not significant for the PrL-R group (*P* > 0.05, Pearson's product moment correlation, *n* = 10) but was significant for the DP-R group (*P* < 0.001, Pearson's product moment correlation, *n* = 30). The modal frequency of the field recording was calculated with the FFT of the field trace. The IPSP modal frequency was calculated with the NRI method. *Significant difference.

Although rhythmic IPSPs recorded at −30 mV were recorded in both PrL and DP, marked regional differences were observed (Fig.8). Recordings in PrL showed that approximately half of all cells (12/22) received non-rhythmic IPSPs (Fig.8*A*) while the remaining cells (10/22) were rhythmic. In contrast all pyramidal cells recorded (*n* = 30) in the DP region exhibited highly rhythmic IPSPs (Fig.8*C*). Overall IPSPs in the DP were significantly more rhythmic and had greater phase coherence than IPSPs in the PrL region (Fig.8*D* and *E*, Table 2). The amplitude of IPSPs also varied, with significantly larger IPSPs recorded in the DP region (Fig.8*F*, Table 2). In addition, not all the rhythmic IPSPs recorded in the PrL were at the same frequency as the field oscillations, as although some IPSPs were deemed to be rhythmic, the frequency of the IPSPs was considerably slower than the concurrently recorded field oscillation (Fig.8*G*). In contrast in the DP region all cells had IPSPs at the same modal frequency as the field oscillations (Fig.8*H*). These data demonstrate that all DP cells exhibited large rhythmic IPSPs that could contribute to the larger area power of the oscillations reported above in this region.

**Table 2:** IPSP coherence and amplitude values in the PrL and DP regions

IPSP			
groups	NRI	log*Z*	Amplitude (mV)
PrL - R (*n* = 10)	0.37 ± 0.1*	3.9 IQR 3.4–4.8*	4.8 IQR 3.5–6.9
PrL – NR (*n* = 12)	−	3.4 IQR 2.7–3.9*	3.1 IQR 2.6–3.6*
PrL – All (*n* = 22)	−	3.6 IQR 3.1–4.1**	3.5 IQR 2.9–4.9**
DP - R (*n* = 30)	0.75 ± 0.04*	5.3 IQR 5.1–5.4*,**	8.5 IQR 5.5–9.7*,**

Grouped data of NRI, phase coherence (log*Z*) and amplitude values extracted from rhythmic (R), non-rhythmic (NR) IPSPs recorded from PrL and DP cells. *,**Statistical significance. Out of the 22 cells in the PrL only 10 received rhythmic IPSPs but 30 cells in the DP received highly rhythmic IPSPs. Overall, DP cells received stronger, more rhythmic and highly coherent to the field phase inhibitory synaptic events.

## Discussion

In this study we have shown that robust oscillations in the (beta (20–30 Hz) range can be evoked with Cb–KA in three subregions of the mPFC *in vitro* (PrL, IL and DP) as reported previously in neocortical areas (Buhl *et al*. 1998; Yamawaki *et al*. 2008; Oke *et al*. 2010; Anver *et al*. 2011; Raver & Keller 2014) and in the anterior cingulate region of the PFC (Steullet *et al*. 2014). Oscillations in the PrL and DP were dependent on fast GABA_A_ receptors and AMPA receptor activation, as shown in other studies of oscillations in the PFC *in vitro* (McNally *et al*. 2011; Steullet *et al*. 2014) and other cortical areas (e.g. Buhl *et al*. 1998; Fisahn *et al*. 1998; Mann *et al*. 2005; Oke *et al*. 2010; Whittington *et al*. 2011). In addition, while NMDA blockade had no effect in the PrL, it caused a small decrease in the DP region. Several studies have shown that the effects of NMDA antagonists on network oscillations can vary depending upon the method used to evoke oscillations and the region studied (Roopun *et al*. 2008; McNally *et al*. 2011).

The oscillations recorded here in the PrL and DP have a slower frequency than those evoked with Cb–KA in somatosensory cortex (Buhl *et al*. 1998), although that study did use mice we feel this frequency difference represents a genuine subregional difference. Several studies (van Aerde *et al*. 2008; McNally *et al*. 2011; Stuellet *et al*. 2014) have shown PFC oscillations ranging from 11 Hz to 60 Hz, using either Cb or KA alone or combined, suggesting that this region is capable of generating more complex patterns of oscillations than are seen *in vitro* in the somatosensory cortex. In addition, one study using combined Cb–KA (Raver & Keller 2014) recorded mixed beta or gamma frequency activity in the PFC (subregion not identified) but only gamma in the somatosensory cortex.

The slower frequency of oscillations generated in this region (20–30 Hz) with Cb–KA compared to largely gamma (∼30–40 Hz) activity recorded in sensory cortical areas (Buhl *et al*. 1998) may reflect the role of the mPFC in integrating multiple sources of input from a wide variety of brain regions (Heidbreder & Groenewegen 2003; Kesner & Churchwell 2011). Beta frequency oscillations have been implicated in long-range communication while gamma frequency activity is proposed to play more of a role in local processing (Kopell *et al*. 2000, 2014; Engel & Fries 2010; Donner & Siegel 2011). Although our data are dominated by beta frequency activity, PrL circuits (like other cortical areas) are capable of generating a range of different frequencies of network activity under different conditions both *in vitro* (van Aerde *et al*. 2008; McNally *et al*. 2011) and *in vivo* (Fujisawa *et al*. 2008; Brockmann *et al*. 2011; Gardner *et al*. 2013).

### Subregional differences between PrL and DP

Although both the PrL and DP regions generated similar frequency oscillatory activity, the oscillations, as discussed above, were larger in the DP region than PrL. The area power difference persisted when slices were trimmed, although in separated PrL slices oscillations were slightly faster, suggesting a possible interaction between PrL and DP similar to that reported between PrL and IL (van Aerde *et al*. 2008). The low phase coherence between PrL and DP suggests volume conduction is not an issue in the intact slices and the data from trimmed slices demonstrates each region was capable of generating oscillations independently.

In addition to a difference in the power of the oscillations between PrL and DP, we have identified a number of cellular differences in pyramidal neurons in the two regions. The main pyramidal cell type is so-called regular spiking (RS) cells either with, or without, evidence of an *I*_h_ activated by hyperpolarising steps (Wang *et al*. 2007; Dembrow *et al*. 2010; Gee *et al*. 2012). We found that in the absence of network oscillations, pyramidal cells in the DP region had more depolarised resting membrane potentials at ∼−65 mV compared to ∼−74 mV in the PrL region. The greater proportion of cells with burst firing properties, lower firing thresholds and more depolarised membrane potentials in the DP region could all contribute to the larger oscillatory area power we observed in DP after application of Cb–KA, as less depolarising drive would be required to activate the network.

More recently PFC pyramidal cells have been divided into two broad classes based on their connectivity: RS cells with *I*_h_ project subcortically and RS cells with little or no *I*_h_ project intracortically (Dembrow *et al*. 2010). We also found that pyramidal cells could be divided into two broad categories but the proportions of pyramidal cells with *I*_h_ varied between dorsal and ventral PFC regions. In our study 94% of recorded presumed pyramidal cells in the DP had *I*_h_ compared to 47% in PrL. However, the situation may be even more complex, as a recent study combining detailed electrophysiological recordings and morphological analysis, suggest an even greater diversity of principal cell types, with one to four different pyramidal cell types reported in each layer (I–VI) of the PrL region (van Aerde & Feldmeyer 2013).

As outlined above network oscillations in both PrL and DP were dependent upon GABA_A_ and AMPA receptor activation, suggesting similar mechanisms may underlie rhythm generation in mPFC to those described for pyramidal-interneuron network gamma (PING) in the hippocampus and superficial layers of the neocortex (Whittington *et al*. 2011). Although we found most mPFC pyramidal cells fired with a low average firing rate (<10 Hz) many cells fired rhythmically. R_slow_ cells tended to fire rhythmically at ∼5 Hz, within the theta frequency range (4–12 Hz), and these cells probably corresponded to cells described previously in the mPFC that have intrinsic subthreshold membrane potential oscillations in this frequency range (Yang *et al*. 1996). R_fast_ cells fired more coherently at the peak negativity of the field oscillation that coincides with the decay phase of the IPSP recorded intracellularly. There was a much higher proportion of R_fast_ cells in DP (56%) compared to PrL (25%), which suggests that more cells in the DP are firing highly coherently with the field oscillation, which could provide a greater excitatory input to neighbouring pyramidal cells and interneurons, resulting in the larger field oscillation power in the DP region.

Synaptic potentials were recorded in pyramidal cells held at −30 mV during the oscillations in PrL and DP. Although these PSPs will have contained mixed EPSP–IPSPs several lines of evidence suggest these potentials were dominated by the IPSPs: there was a 180 deg phase difference between the field and IPSPs, cells fired spikes preferentially at the peak negativity of the field oscillation and recorded EPSPs were not coherent with the oscillation. In addition, we found a significant correlation between the area power of the field oscillation and the presumed IPSP amplitude, in agreement with a study in the hippocampus suggesting that the IPSP makes the largest contribution to the extracellular field oscillation (Oren *et al*. 2010).

However, although presumed IPSPs were recorded in cells at −30 mV in both the PrL and DP there were significant differences between the regions. In the DP region all cells received rhythmic IPSPs at the same frequency as the on-going field oscillation. In contrast in the PrL 12/22 cells received rhythmic IPSPs, but in the remainder of cells IPSPs were non-rhythmic. The NRI, phase coherence and IPSP amplitude were all significantly greater in DP when compared with PrL, which, as stated above, probably contributed to the larger area power of oscillations seen in the DP region. Using optogenetic techniques Lee *et al*. (2014) recently showed that the pyramidal cells in PrL and IL with *I*_h_ were preferentially inhibited by parvalbumin (PV) positive interneurons, resulting in greater feed-forward inhibition in these cells. If this were also the case in the DP region then we would expect greater PV-mediated inhibition in DP where the vast majority of cells had *I*_h_ that, in turn, might account for the large rhythmic IPSPs we observed in the DP region. Although a few studies have characterized FS interneuron properties in the PFC (e.g. Tranthan-Davidson *et al*. 2004; Povysheva *et al*. 2008, 2013; Zaitsev & Lewis 2013) further studies detailing different interneuron subtypes in mPFC, and their role in different network oscillations are still required.

### The role of DP

Currently little is known about the role of the DP and, to our knowledge, this is the first study to demonstrate robust oscillations in this region *in vitro*. If considered at all in the past, DP has been grouped with IL cortex and considered part of the ventral PFC involved in visceral and autonomic functions (Heidbreder & Groenewegen 2003). However, the DP occupies a large area in the rostral–caudal direction in the PFC above the tenia tecta (Paxinos & Watson 2007). Two recent anatomical studies have described in detail a distinct connectivity of the DP (Akhter *et al*. 2014; Zingg *et al*. 2014). These studies have shown that DP receives inputs from the posterior insula cortex and lateral entorhinal cortex (Zingg *et al*. 2014) and that its connectivity is distinct from the IL (Akhter *et al*. 2014), suggesting DP and IL mediate separate PFC functions. The specific role of DP and its interaction with other regions of the PFC requires further study.

In conclusion, we have shown important subregional differences in fast network oscillations in the mPFC that, we propose, are due to differences in the cellular components within each network, and may reflect the different functions of each mPFC subregion.

## Abbreviations

ACC: anterior cingulate
ACSF: artificial cerebrospinal fluid
AMPA: α-amino-3-hydroxy-5-methyl-4-isoxazolepropionic acid
ANOVA: analysis of variance
BF: burst firing
Cb: carbachol
d-AP5: dl-2-amino-phosphonopentanoic acid
DP: dorsopeduncular
EPSPs: excitatory postsynaptic potentials
FFT: fast Fourier transform
FS: fast-spiking
GABA: γ-aminobutyric acid
KA: kainate
*I*_h_: cAMP-dependent hyperpolarisation activated current
IL: infralimbic
IFF: inter-spike firing frequency
IPSPs: inhibitory postsynaptic potentials
IQR: interquartile range
LFP: local field potential
mPFC: medial prefrontal cortex
NMDA: *N*-methyl-d-aspartate
NR: non-rhythmic
NRI: neuronal rhythmicity index
PFA: paraformaldehyde
PFC: prefrontal cortex
PrL: prelimbic
pmf: probability mass function
PSD: power spectral density
PSP: post-synaptic potential
PV: parvalbumin
RI: rhythmicity index
RM-ANOVA: repeated measures analysis of variance
RS: regular spiking
SEM: standard error of the mean
